# Clinical Application of iNKT Cell-mediated Anti-tumor Activity Against Lung Cancer and Head and Neck Cancer

**DOI:** 10.3389/fimmu.2018.02021

**Published:** 2018-09-07

**Authors:** Mariko Takami, Fumie Ihara, Shinichiro Motohashi

**Affiliations:** ^1^Department of Medical Immunology, Graduate School of Medicine, Chiba University, Chiba, Japan; ^2^Department of Otorhinolaryngology, Head and Neck Surgery, Graduate School of Medicine, Chiba University, Chiba, Japan

**Keywords:** antigen presenting cells (APCs), invariant natural killer T cells (iNKT cells), cancer immunotherapy, non-small cell lung cancer (NSCLC), head and neck cancer (HNC)

## Abstract

Invariant natural killer T (iNKT) cells produce copious amounts of cytokines in response to T-cell receptor (TCR) stimulation by recognizing antigens such as α-galactosylceramide (α-GalCer) presented on CD1d; thus, orchestrating other immune cells to fight against pathogen infection and tumors. Because of their ability to induce strong anti-tumor responses and the convenience of their invariant TCR activated by a synthetic ligand, α-GalCer, iNKT cells have been intensively studied for application in immunotherapeutic approaches to treat cancer patients in the clinic. Here, we summarize the clinical trials of iNKT cell based immunotherapy for non-small cell lung cancer, and head and neck cancer. Although solid tumors are thought to be refractory to immunotherapeutic approaches, our clinical trials showed that the intravenous injection of α-GalCer-pulsed antigen presenting cells (APCs) activated endogenous iNKT cells and iNKT cell dependent responses. Moreover, an increase in the number of IFN-γ producing cells in PBMCs was associated with prolonged survival. The marked infiltration of iNKT cells and the accumulation of conventional T cells in the tumor microenvironment were also observed after the administration of α-GalCer-pulsed APCs and/or *ex vivo* activated iNKT cells. In cases of advanced head and neck squamous cell carcinoma, the increased accumulation of iNKT cells in the tumor microenvironment was correlated with objective clinical responses. We will also discuss potential combination therapies of iNKT cell based immunotherapy to achieve enhanced anti-tumor activity and provide better treatment options for these patients.

## Introduction

Invariant natural killer T (iNKT) cells comprise 1–2% of the mouse spleen and less than 0.1% in human peripheral tissues (1). Although a small population, iNKT cells elicit strong immune responses by producing large amounts of cytokines, which lead to other immune cell responses as well as inducing cytotoxicity; thus, they have an important role in both innate and adaptive immunity.

Whereas conventional T cells express diverse T cell receptors (TCR) after TCR rearrangement and recognize their cognate peptide presented on MHC, iNKT cells express monoclonal TCRs composed of a Vα24-Jα18 chain and Vβ11 chain in humans, and a Vα14-Jα18 chain and Vβ8.2 chain in mice that recognize glycolipid antigens presented on CD1d, a MHC class I like molecule (2, 3). Another unique characteristic of iNKT cells that distinguishes them from conventional T cells is the expression of NK cell receptors including CD56, CD16, NKG2D, and CD161 (4). Therefore, iNKT cells recognize non-self in a TCR dependent manner as well as by NK cell receptors. In 1997, Kawano et al. identified α-galactosylceramide (α-GalCer), derived from the marine sponge, as a glycolipid ligand that activates iNKT cells and this discovery has enhanced our understanding of the function and role of iNKT cells in immunity over the past 20 years (5). α-GalCer is a potential therapeutic tool to control immune responses in a variety of disease conditions such as cancer and autoimmunity.

In tumor immunity, the role of iNKT cells was initially demonstrated by using mouse models. CD1d knockout mice or Vα14 knockout mice, which lack iNKT cells, are more susceptible to tumors compared with wild type mice, suggesting that iNKT cells play a crucial role in anti-tumor immunity. Because iNKT cells recognize α-GalCer presented on CD1d expressed on antigen presenting cells (APCs), Toura et al. demonstrated that the administration of α-GalCer loaded dendritic cells (DCs) expanded NKT cells and rejected tumors in a mouse liver metastasis and lung metastasis model (6). Furthermore, even established metastatic tumors were rejected by the administration of α-GalCer loaded DCs. iNKT cells produce large amounts of cytokines including IFN-γ and IL-4 upon TCR stimulation by recognizing α-GalCer presented on CD1d. These events consequently lead to the activation of NK cells and CD8^+^ T cells and the conversion of immature DCs to mature DCs; thus, enhancing other immune cell responses to indirectly eradicate tumors (7). iNKT cells also produce granzyme A and granzyme B and express Fas ligand and TRAIL to exert a direct tumor killing effect (8). These pleiotropic functions of iNKT cells are thought to be involved in tumor eradication. Based on these findings, we applied α-GalCer loaded DCs as a tool for iNKT cell based immunotherapy to treat cancer patients.

Immunotherapy is widely recognized as a powerful cancer treatment tool because immune checkpoint blockade with programmed death-1 (PD-1) antibody eradicated tumors dramatically in some patients with lung cancer (9). Since then, a variety of immunotherapeutic approaches has been studied to determine better treatment options for cancer patients. One of these approaches is iNKT cell based therapy and clinical trials of iNKT cell-targeted immunotherapy for lung cancer, and head and neck cancer patients have been conducted at Chiba University. Herein, we will review our clinical trials of iNKT cell based immunotherapy (Table 1). We will also discuss the next step of iNKT cell based immunotherapy including combination therapies and induced pluripotent stem (iPS) cell-derived iNKT cells.

**Table 1:** iNKT cell based immunotherapy.

**Cancer type**	**Number of patients (completed/enrolled)**	**Phase**	**Treatment (injection)**	**Number of cells injected**	**Number of injection**	**Immunological responses**	**Clinical outcomes**	**References**
NSCLC	9/11	I	α-GalCer-pulsed APCs	Level 1: 5 × 10^7^ cells/m^2^ Level 2: 2.5 × 10^8^ cells/m^2^ Level 3: 1 × 10^9^ cells/m^2^	4	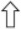 iNKT cell number 3/3 patients (level 3)		(10)
	17/23	I-II	α-GalCer-pulsed APCs	1 × 10^9^ cells/m^2^	4	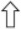 iNKT cell number 6/17 patients 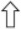 IFN-γ producing cell number 10/17 patients		(11)
	4/4	(–)	α-GalCer-pulsed APCs	1 × 10^9^ cells/m^2^	1	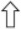 % of iNKT cells in TILs		(12)
	6/6	I	iNKT cells	Level 1: 1 × 10^7^ cells/m^2^ Level 2: 5 × 10^7^ cells/m^2^	2	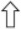 iNKT cell number 2/3 patients (level 2) 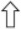 IFN-γ producing cell number 3/3 patients (level 2)		(13)
HNC	9/9	I	α-GalCer-pulsed APCs	1 × 10^8^ cells/m^2^	2	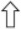 iNKT cell number 4/9 patients 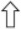 IFN-γ producing cell number 8/9 patients	PR 1/9 SD 5/9 PD 3/9	(14)
	8/8	I	α-GalCer-pulsed APCs iNKT cells	1 × 10^8^ cells/m^2^ 5 × 10^7^ cells/m^2^	2 1	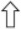 iNKT cell number 7/8 patients 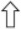 IFN-γ producing cell number 7/8 patients	PR 3/8 SD 4/8 PD 1/8	(15)
	10/10	II	α-GalCer-pulsed APCs iNKT cells	1 × 10^8^ cells/m^2^ 5 × 10^7^ cells/m^2^	1 1	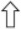 iNKT cell number 9/10 patients 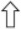 IFN-γ producing cell number 10/10 patients	PR 5/10 SD 5/10	(16)

### iNKT cell based immunotherapy for lung cancer

Lung cancer is a leading cause of cancer death worldwide. Non-small cell lung cancer (NSCLC) is the most common type of lung cancer and is classified as one of three representative subtypes: squamous cell carcinoma, adenocarcinoma, and large cell carcinoma. Although cytotoxic chemotherapeutic agents have been used as the first line treatment for advanced or unresectable NSCLC patients for many years, some PD-1 signaling blockade antibodies have been clinically approved and are used as a first line or second line treatment in recent years (17). These treatments have had impressive outcomes for some, but not all, NSCLC patients; thus, alternative therapies are still highly desired to improve the prognosis of NSCLC patients.

### Establishment of α-GalCer-loaded APCs

In general, DCs are generated from peripheral blood CD14^+^ monocytes cultured in the presence of DC inducing cytokines, GM-CSF and IL-4. Some clinical studies using these cells to target cytotoxic T lymphocytes have been performed (18, 19). However, these culture methods might not be suitable for iNKT cell based clinical trials because a large number of DCs are required to encounter and activate the small population of iNKT cells in the periphery of patients. Therefore, we developed a new method to obtain a large number of APCs from peripheral blood mononuclear cells (PBMCs). In addition to DCs, other immune cells such as activated T cells express CD1d on their cell surface; therefore, the potential use of cultured whole PBMCs instead of DCs was suggested (20). It was reported that PBMCs cultured in the presence of GM-CSF and IL-2 presented α-GalCer on CD1d and induced the expansion and activation of human iNKT cells better than DCs generated in the presence of GM-CSF and IL-4.

### Early phase clinical trials: administration of α-GalCer-loaded APCs

Because a large number of APCs can present α-GalCer and activate iNKT cells more effectively compared with monocyte derived DCs, and can be generated from PBMCs by culturing with GM-CSF and IL-2, we designed a phase I clinical trial for NSCLC patients using α-GalCer-pulsed APCs (10). Eleven patients with stage IV or recurrent NSCLC were enrolled in this study and nine completed the treatment. In this phase I study, safety profiles of α-GalCer-pulsed APCs were examined at three different doses at level 1: 5 × 10^7^, level 2: 2.5 × 10^8^ and level 3: 1 × 10^9^ cells/m^2^. Patients received four intravenous injections of α-GalCer-pulsed APCs over 3 months. While objective clinical responses were not observed in all cases, patients who received the level 3 dose of α-GalCer-pulsed APCs exhibited iNKT cell expansion in the periphery and showed long term survival for over one year. These iNKT cells had high *ifn-*γ mRNA expression. No severe adverse events over grade II were observed in all cases including the highest dose at level 3, suggesting α-GalCer-pulsed APC administration was safe and a feasible treatment. Moreover, the level 3 dose (1 × 10^9^ cells/m^2^) was considered the most effective and safe dose of α-GalCer-pulsed APCs to treat advanced NSCLC patients.

We then designed the next step, a phase I-II clinical trial for NSCLC patients, to extend the study of iNKT cell based immunotherapy (11). Twenty-three patients were enrolled in this study from February 2004 to August 2006, and 17 completed the protocol treatment. Patients were either stage IIIB, stage IV or recurrent NSCLC patients who received standard therapy. The safety of α-GalCer-pulsed APCs and immunological responses were examined at a dose of 1 × 10^9^ cells. All patients received two courses with four injections of 1 × 10^9^ α-GalCer-pulsed APCs. Regarding adverse events, one patient experienced the recurrence of deep vein thrombosis (DVT) (estimated as a grade III adverse event) and required hospitalization for the continuous injection of heparin. The Chiba University Quality Assurance Committee on Cell Therapy determined no clear relationship between the cell therapy and DVT. Because no severe adverse effects were observed in other patients, the safety of the administration of α-GalCer was confirmed. Regarding immune monitoring, 10 patients had a greater than two-fold increase of IFN-γ producing cells in the periphery after the administration of α-GalCer-pulsed APCs (good responders) whereas seven patients showed mild or no increase of IFN-γ producing cells (poor responders). Moreover, the increase of IFN-γ producing cells in the periphery of patients correlated with the median survival time (MST) and good responders showed a longer MST (29.3 months) compared with poor responders (9.7 months). Overall, the MST of all cases was 18.6 months. Because this clinical trial was not a randomized controlled study, we could not conclude the superiority of α-GalCer-pulsed APCs; however, these data encouraged us to perform a comparative study to compare the α-GalCer-pulsed APCs and standard cytotoxic drugs.

To further elucidate the mechanisms by which the administration of α-GalCer-pulsed APCs trigger iNKT cell immune responses in the tumor microenvironment of NSCLC patients, we performed a clinical study targeting four patients diagnosed as stage IIB and IIIA NSCLC who underwent surgical treatment compared with 6 patient controls (12). Patients received a single intravenous injection of 1 × 10^9^ α-GalCer-pulsed APCs seven days prior to surgery and characteristic tumor infiltration was analyzed from surgically removed tumor tissue specimens. A higher percentage of iNKT cells was observed in tumor infiltrating lymphocytes (TIL) compared with mononuclear cells isolated from normal lungs and draining lymph nodes. The percentage of iNKT cells in TIL was increased with α-GalCer-pulsed APC administration compared with control groups. Moreover, increased numbers of IFN-γ producing cells in TIL were observed after α-GalCer-pulsed APC administration, indicating that the systemic intravenous administration of α-GalCer-pulsed APCs led to local iNKT cell accumulation in the tumor microenvironment and induced immune responses by producing IFN-γ.

### Phase I clinical trial: administration of iNKT cells

To increase iNKT cell numbers in the periphery, the administration of *in vitro* expanded NKT cells was performed as a phase I clinical trial in six patients with recurrent lung cancer (13). iNKT cells were prepared *in vitro* from PBMCs cultured in the presence of α-GalCer and IL-2. *In vitro* expanded iNKT cells (level 1: 1 × 10^7^ cells, level 2: 5 × 10^7^ cells per injection) were intravenously transferred to patients. Whereas it was previously reported that iNKT cells in cancer bearing patients had a lower frequency and impaired proliferation capability, iNKT cells derived from patients in this study expanded and produced Th1 dominant cytokines including IFN-γ along with tumoricidal activity *ex vivo*. No patients had severe adverse events during the study. Two of three patients who received a level 2 dose showed increased IFN-γ production while no patients met the criteria for an objective clinical response. These results suggested that the administration of α-GalCer-pulsed APCs was a more effective treatment compared with the administration of *ex vivo* expanded iNKT cells.

The intravenous injection of α-GalCer-pulsed APCs in patients with advanced or recurrent NSCLC after first line treatment was accepted as an advanced medicine by the Japanese Ministry of Health, Labour and Welfare in 2011. Since then, 35 patients were enrolled in this study and 32 received all courses of treatment. The follow-up was completed in 2017. We are currently analyzing the clinical efficacy and immune responses.

### iNKT cell based immunotherapy for head and neck cancer

Head and neck cancer (HNC) accounts for about 5% of all cancers. Despite the development of multidisciplinary treatment involving surgery, radiotherapy, and chemotherapy for advanced cases, the recurrence rate is still high; thus, the survival rate remains relatively low. Moreover, the quality of life (QOL) of patients who receive these combination therapies is often severely impaired. To improve the prognosis and QOL of patients with head and neck cancer, the development of new therapies is highly desired. Because iNKT cell based immunotherapy for NSCLC patients showed promising results in the treatment of solid tumors, we designed clinical studies of iNKT cell based immunotherapy for HNC patients. While the intravenous administration of α-GalCer-pulsed APCs was used in our clinical trials for NSCLC patients, we found that nasal submucosa injection induced APCs to migrate to the neck lymph node area (21). Furthermore, the nasal submucosa injection of α-GalCer-pulsed APCs increased the number of iNKT cells and IFN-γ producing cells in the peripheral tissues of patients (22). In contrast, the injection of α-GalCer-pulsed APCs into the oral floor submucosa induced tolerance with increased numbers of CD45RA^−^Foxp3^high^ Tregs instead of anti-tumor activity. These results indicated that the administration of α-GalCer-pulsed APCs via the nasal submucosa was a better option for HNC patients. We also confirmed that the number and function of iNKT cells were not affected by radiation therapy, suggesting that iNKT cell based immunotherapy might be an adjuvant treatment of radiation therapy for advanced HNC patients (23).

### Clinical trials of iNKT cell based immunotherapy for patients with advanced and recurrent HNC

We conducted a phase I clinical trial study of iNKT cell based immunotherapy for patients with recurrent or unresectable HNC using α-GalCer-pulsed APCs (14). Nine patients were enrolled in this study and α-GalCer-pulsed APCs (1 × 10^8^ cells/injection) were administrated into the nasal submucosa. During the study period, no serious adverse events over grade 3 were observed. Moreover, the number of peripheral iNKT cells increased in four patients and an increase in IFN-γ producing cells was observed in eight patients. These results suggested that the administration of α-GalCer-pulsed APCs into the nasal submucosa was a safe and effective approach to induce iNKT cell anti-tumor responses. However, the clinical efficacy was not satisfactory in this study.

To improve clinical efficacy and confirm the safety of iNKT cell based immunotherapy in patients with recurrent or unresectable HNC, we conducted a combination therapy with *ex vivo* expanded iNKT cells and α-GalCer-pulsed APCs. We performed a phase I study of eight patients with recurrent HNC that were refractory to standard therapy (15). Because most HNCs receive blood supply through terminal arteries, the intra-arterial infusion of anti-cancer agents is commonly used (24). Patients received two nasal submucosa injections of α-GalCer-pulsed APCs (1 × 10^8^ cells/injection) and one intra-arterial infusion of activated iNKT cells (5 × 10^7^ cells/injection). No patients showed severe adverse events except one patient who had partial response (PR) with pharynx-skin fistula (grade III). PR was observed in three patients while stable disease (SD) or progressive disease (PD) was observed in four patients. The iNKT cell number in the periphery was increased over 3-fold after the treatment in all patients and increase in IFN-γ producing cells in the periphery was over 4-fold in seven patients (15).

In a phase II study, 10 patients with locally recurrent HNC and were indicated for salvage surgery were enrolled. Therefore, *ex vivo* expanded iNKT cells (5 × 10^7^ cells) were administrated via the terminal artery and α-GalCer-pulsed APCs (1 × 10^8^ cells) were administrated into the nasal submucosa. No severe adverse events over grade 2 were observed in any patients (16). The number of iNKT cells in the periphery was increased in nine patients. Furthermore, IFN-γ producing cells in the periphery were increased in all patients. Additionally, five patients achieved PR and five patients achieved SD. Interestingly, the percentage of infiltrated iNKT cells was higher in PR cases compared with SD cases. Based on these results, we confirmed that the co-administration of *ex vivo* expanded iNKT cells and α-GalCer-pulsed APCs augmented both anti-tumor immune responses and clinical efficacy.

### Ongoing advanced medicine for HNC patients

A double-blinded randomized study is essential to provide compelling evidence to develop novel therapies for cancers; thus, we conducted a double-blinded randomized study of α-GalCer-pulsed or non-pulsed APC administration to the nasal submucosa in patients with HNC who archived complete response (CR) after standard therapy. The aim of the study was to clarify the role of iNKT cells to prevent the recurrence of HNC after standard treatment in those thought to have minimal residual disease. This study was accepted as advanced medical care by the Japanese Ministry of Health, Labour and Welfare in 2012. Patients diagnosed with stage IV HNC and who achieved CR after standard therapy were enrolled in this study to investigate the prevention of the recurrence of HNC. Patients received two α-GalCer-pulsed APC injections (1 × 10^8^ cells/injection) into the nasal submucosa on days seven and ten. Patient recruitment, follow-up and immunological analysis are in progress.

## Future direction

While iNKT cell based immunotherapy is considered a promising treatment for patients with lung cancer, and head and neck cancer based on the results of previous clinical trials mentioned above, some factors can be improved to provide better treatment options for these patients. Because iNKT cells are such a small population of lymphocytes, it is critical to determine how to efficiently increase the iNKT cell number and enhance their function including IFN-γ production and tumoricidal activity. We are currently investigating two approaches to make our current iNKT cell based immunotherapy more effective. One is to generate iPS cell derived NKT cells (iPS-NKT cells), and the other is combination therapy.

iPS cells are generated from somatic cells by introducing genes to express four transcription factors (*Oct4, Sox2, Klf4*, and *c-Myc*) named Yamanaka factors (25). They are reprogrammed to an embryonic stem like cell, which can self-renew and differentiate into multiple cell types. Yamada et al. successfully generated human iPS-NKT cells from iNKT cells isolated from peripheral blood mononuclear cells or cord blood mononuclear cells by introducing Yamanaka factors (26). iPS-NKT cells showed an iPS cell phenotype that was capable of proliferating and that retained NKT cell functions such as cytokine production and cytotoxic activity. The generation of iPS-NKT cells will help acquire sufficient numbers of iNKT cells from patients with low numbers of iNKT cells for next generation iNKT cell based immunotherapy. We are currently performing nonclinical tests of iPS-NKT cells to confirm their safety and efficacy.

Furthermore, we are also investigating potential combination therapies to enhance iNKT cell based immunotherapy. We found that the blockade of PD-1/PD-ligand 1(PD-L1) signaling, an immune checkpoint pathway, enhanced iNKT cell function including cytokine production and tumoricidal activity, suggesting that combined immune checkpoint inhibitor and iNKT cell based immunotherapy might have synergistic effects and exert powerful anti-tumor immunity (27).

## Author contributions

All authors listed have made substantial contributions to the manuscript and have approved it for submission.

### Conflict of interest statement

The authors declare that the research was conducted in the absence of any commercial or financial relationships that could be construed as a potential conflict of interest.
